# Supplementary material for: How the window of visibility varies around polar angle
Source: bioRxiv. 2024 Jul 16:2024.07.12.603257. Preprint. [Version 1] doi: 10.1101/2024.07.12.603257 (PMC11275830; doi:10.1101/2024.07.12.603257)
Supplement: 1 [file NIHPP2024.07.12.603257V1-supplement-1.pdf]

bioRxiv preprint doi: https://doi.org/10.1101/2024.07.12.603257; this version posted July 16, 2024. The copyright holder for this preprint (which was not certified by peer review) is the author/funder, who has granted bioRxiv a license to display the preprint in perpetuity. It is made available under aCC-BY 4.0 International license.

Supporting Information

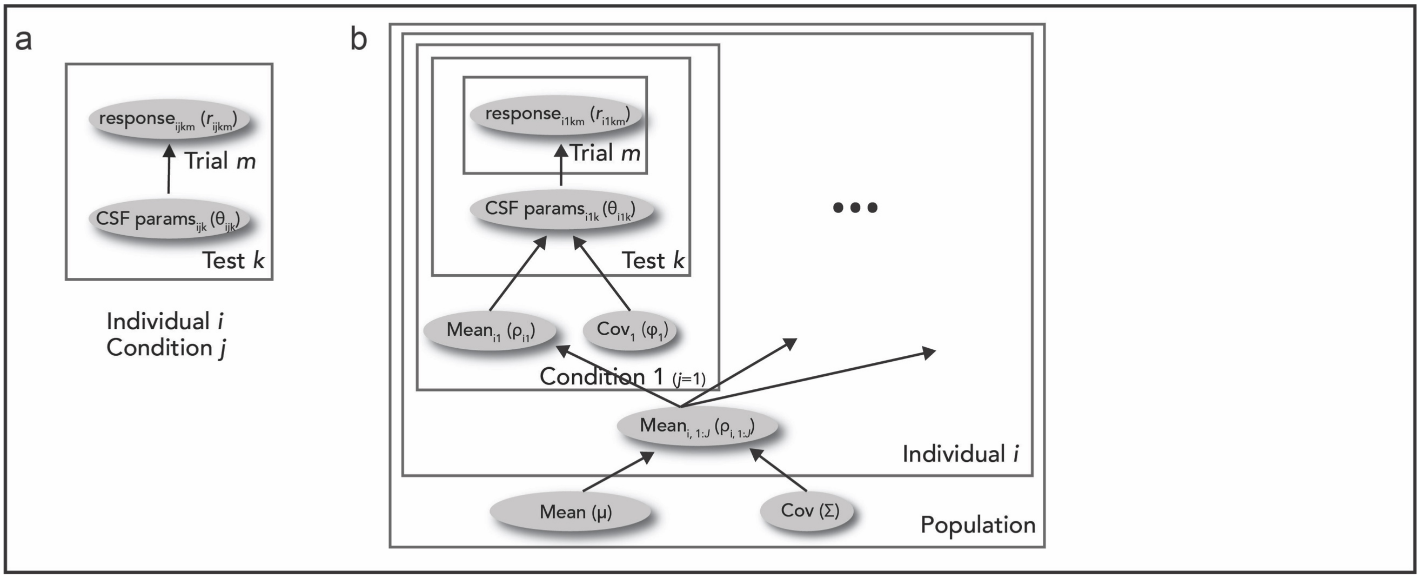

Figure S1. CSF model fitting. (a) Schematic representation of the BIP (Bayesian Inference Procedure). The first stage of the data analysis consists of fitting trial-by-trial data points with the BIP to estimate the CSF parameters – peak-CS, peak-SF, and bandwidth. The BIP computes the posterior distribution of the parameters for each test independently. **(b)** Schematic representation of the HBM (Hierarchical Bayesian Model). In the second stage of the data analysis, the BIP outputs are used as the mean and covariance of the prior distributions of the CSF hyperparameters in the HBM. We used a three-level hierarchical model to incorporate potential relations in the CSF parameters across individuals and tests. For details on the BIP and the HBM, see **Methods**.

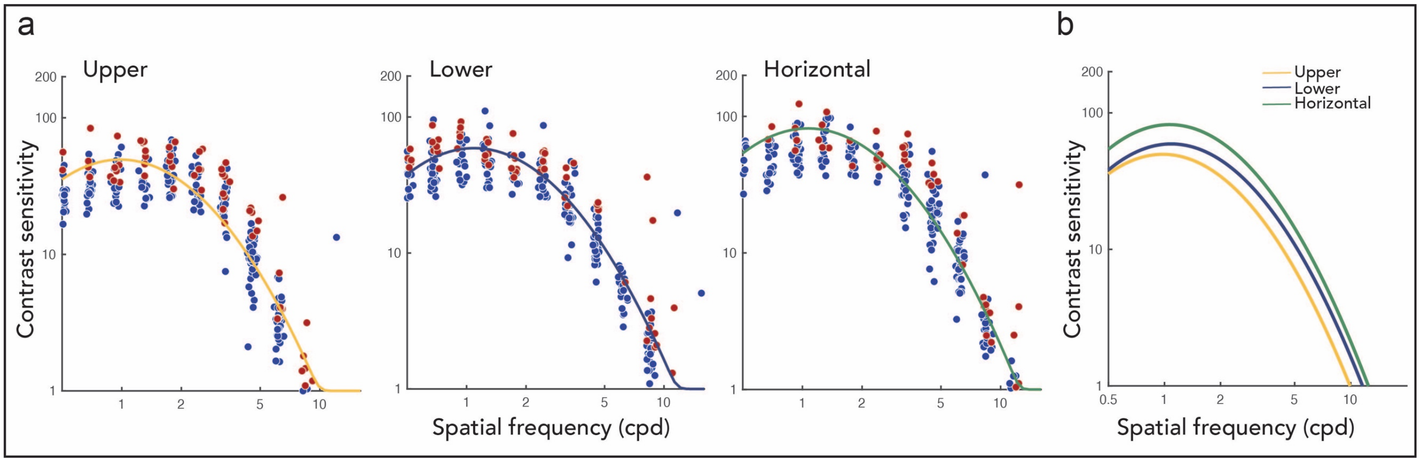

Figure S2. Data from an example observer. (a) Trial-by-trial datapoints (red for incorrect trials, blue for correct trials) and fitted CSFs for the upper, lower, and the horizontal meridians. **(b)** CSFs across locations are overlayed.

bioRxiv preprint doi: https://doi.org/10.1101/2024.07.12.603257; this version posted July 16, 2024. The copyright holder for this preprint (which was not certified by peer review) is the author/funder, who has granted bioRxiv a license to display the preprint in perpetuity. It is made available under aCC-BY 4.0 International license.

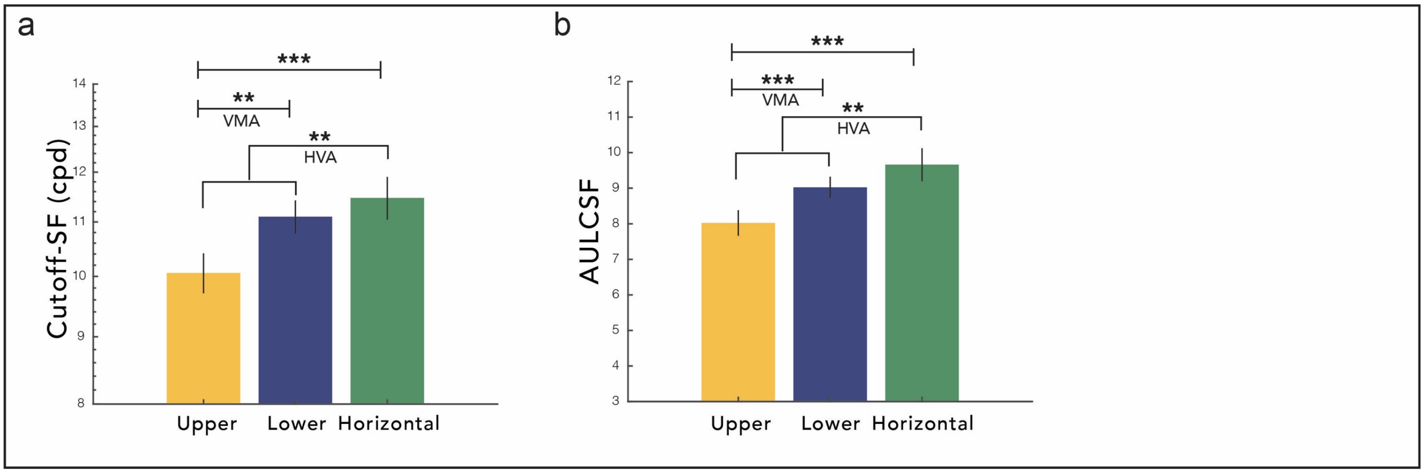

Figure S3. Polar angle sensitivity differences. (a) Cutoff spatial frequency. (b) Area under the log CSF. ***$p < 0.001$, **$p < 0.01$. Error bars are ±1SEM. HVA and VMA denote Horizontal-Vertical Anisotropy and Vertical Meridian Asymmetries, respectively.

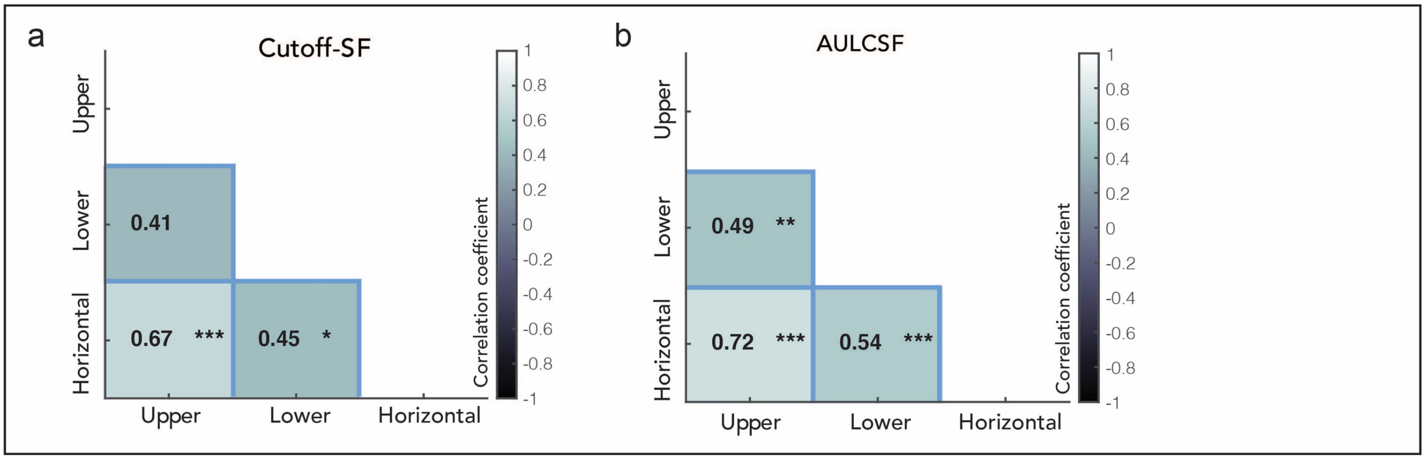

Figure S4. Covariation of each CSF attribute across polar angle locations. (a) Cutoff spatial frequency. (b) Area under the log CSF. Confidence intervals are in **Figure S7**. ***$p < 0.001$, **$p < 0.01$, *$p < 0.05$.

bioRxiv preprint doi: https://doi.org/10.1101/2024.07.12.603257; this version posted July 16, 2024. The copyright holder for this preprint (which was not certified by peer review) is the author/funder, who has granted bioRxiv a license to display the preprint in perpetuity. It is made available under aCC-BY 4.0 International license.

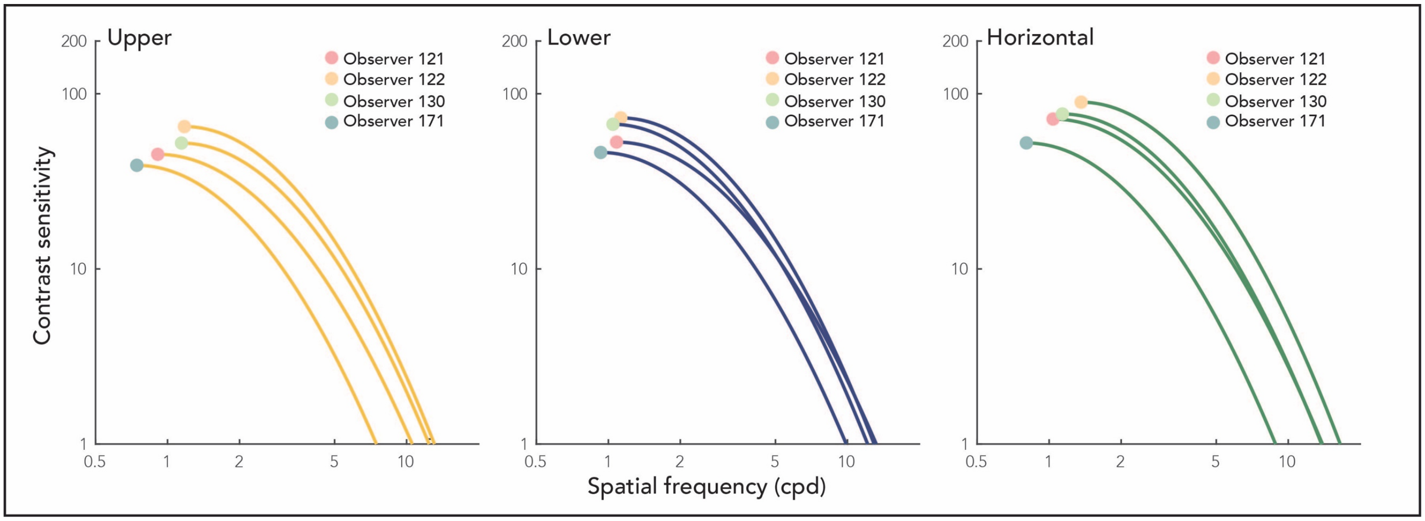

Figure S5. Data from four example observers at each location. Each circled dot indicates the peak-CS and peak-SF of each observer. Note that across locations, observers with higher peak-CS and peak-SF at one location tend to have higher peak-CS and peak-SF at other locations, indicating that CSF attributes are highly correlated across polar angle (**Figure 3**). Moreover, at the horizontal meridian, observers' CSFs are shifted consistently along the diagonal direction in the SF-contrast space (**Figure 4**), where the peak-CS and peak-SF fall closely along a diagonal line. Only the part of the CSF corresponding to spatial frequencies above the peak-SF is shown for a clearer illustration of peak-CS and peak-SF.

bioRxiv preprint doi: https://doi.org/10.1101/2024.07.12.603257; this version posted July 16, 2024. The copyright holder for this preprint (which was not certified by peer review) is the author/funder, who has granted bioRxiv a license to display the preprint in perpetuity. It is made available under aCC-BY 4.0 International license.

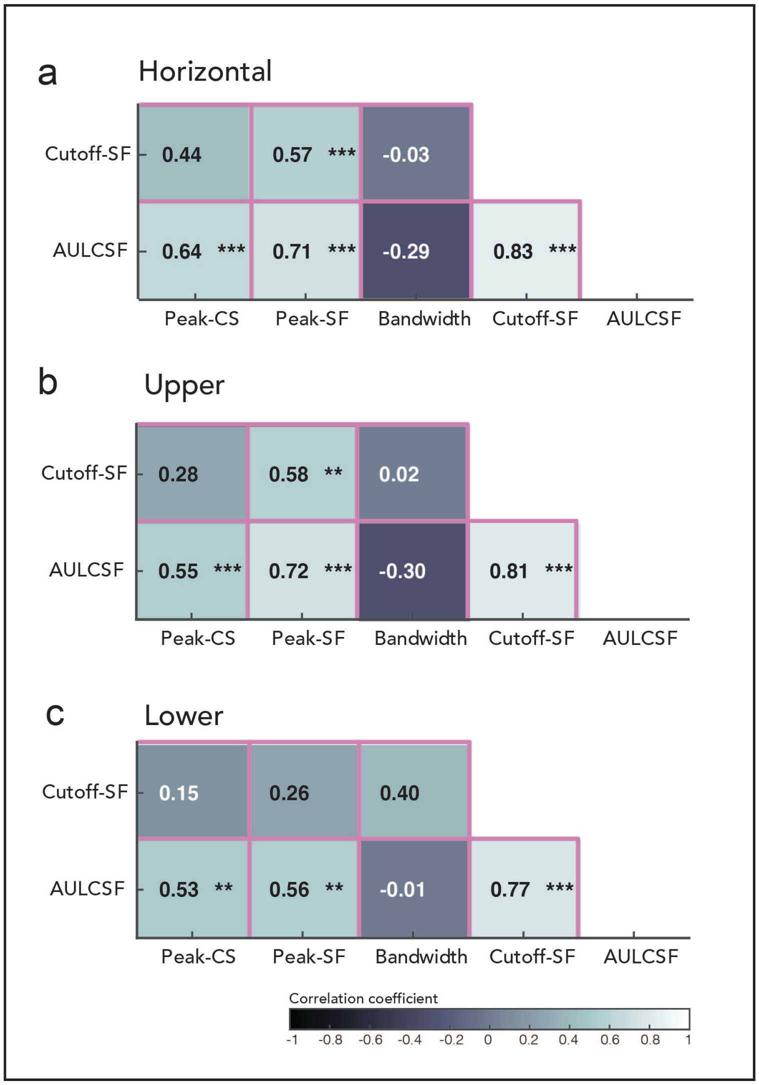

Figure S6. Covariance of CSF attributes within each location (pink cells in **Figure 1d-e**). Correlation between pairs of attributes at the **(a)** Horizontal, **(b)** Upper vertical, and **(c)** Lower vertical meridian. The color of each cell corresponds to the strength of correlation. Each cell corresponds to a pink cell in **Figure 1d**. Confidence intervals are in **Figure S7**. ***p < 0.001, **p < 0.01. Black and white letters are for visibility.

bioRxiv preprint doi: https://doi.org/10.1101/2024.07.12.603257; this version posted July 16, 2024. The copyright holder for this preprint (which was not certified by peer review) is the author/funder, who has granted bioRxiv a license to display the preprint in perpetuity. It is made available under aCC-BY 4.0 International license.

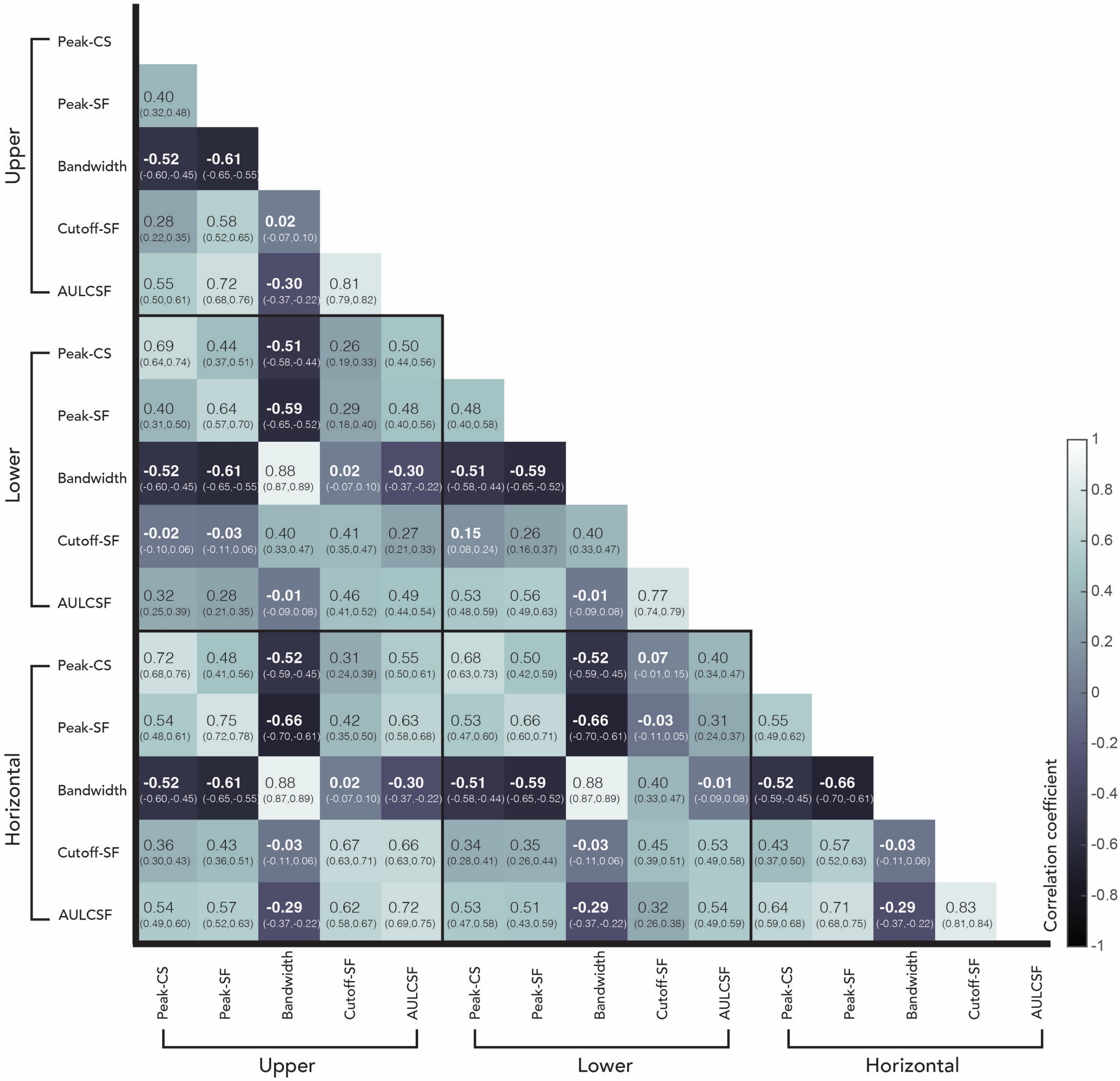

Figure S7. Correlation coefficients and confidence intervals. Correlation coefficients are in the center of each cell, and confidence intervals are in the parentheses below. The distribution of MCMC samples (**Methods**) was used to compute the 68% confidence interval. Black and white letters are for visibility.

bioRxiv preprint doi: https://doi.org/10.1101/2024.07.12.603257; this version posted July 16, 2024. The copyright holder for this preprint (which was not certified by peer review) is the author/funder, who has granted bioRxiv a license to display the preprint in perpetuity. It is made available under aCC-BY 4.0 International license.

Table S1. Coefficient of variation (CoV). For each attribute at each location, we computed the CoV (ratio of the standard deviation to the mean), which is useful for comparing the variation across individuals for each CSF attribute which differs in units.

	Upper	Lower	Horizontal
Peak-CS	0.30	0.25	0.25
Peak-SF	0.23	0.14	0.24
Bandwidth	0.18	0.14	0.20
Cutoff-SF	0.19	0.15	0.20
AULCSF	0.24	0.18	0.25
